# Investigating PCBs and OCPs in Lebanese Yogurt: National Contamination Patterns and Health Risk Assessment

**DOI:** 10.3390/foods14162866

**Published:** 2025-08-19

**Authors:** Sandra Sarkis, Jean Claude Assaf, Mantoura Nakad, Tony Tannous, Mireille Harmouche-Karaki, Khalil Helou, Joseph Matta

**Affiliations:** 1 Department of Nutrition, Faculty of Pharmacy, Ecole Doctorale Sciences et Santé (EDSS), Medical Sciences Campus, Saint Joseph University of Beirut (USJ), Beirut 1100, Lebanon; sandra.sarkis@net.usj.edu.lb; 2 Chemical Engineering Department, Faculty of Engineering, University of Balamand, Koura Campus, Kelhat P.O. Box 100, Lebanon; 3 FOE Dean’s Office, Faculty of Engineering, University of Balamand, Koura Campus, Kelhat P.O. Box 100, Lebanon; 4 Faculty of Arts and Sciences, University of Balamand, Tripoli P.O. Box 100, Lebanon; tony.tannous@balamand.edu.lb; 5 Department of Nutrition, Faculty of Pharmacy, Saint Joseph University of Beirut (USJ), Beirut 1100, Lebanon; mireille.harmouche@usj.edu.lb (M.H.-K.); khalil.helou@usj.edu.lb (K.H.); joseph.matta@usj.edu.lb (J.M.); 6 Industrial Research Institute, Lebanese University Campus, Hadat Baabda, Beirut 1100, Lebanon

**Keywords:** yogurt, persistent organic pollutants, polychlorinated biphenyls, organochlorine pesticides, food safety, risk assessment, sustainability, Lebanon

## Abstract

This study investigates the presence of polychlorinated biphenyls (PCBs) and organochlorine pesticides (OCPs) in 165 yogurt samples collected from farms across 11 Lebanese regions. As the first nationwide assessment of these contaminants in yogurt, it addresses a critical gap in Lebanon’s food safety monitoring. Levels of PCBs, ΣDDTs, ΣHCH, HCB, endosulfan, methoxychlor, and dieldrin were quantified, revealing widespread contamination. A total of 40.6% of samples recorded total PCB concentrations exceeding European maximum residue limits (MRLs), with a national mean of 39.26 ng/g fat. Keserwan, and North and South Lebanon showed the highest PCB contamination, likely linked to thermal power generation activities. For OCPs, mean concentrations of all tested compounds exceeded their respective MRLs across all regions. Levels of ΣDDTs surpassed the MRL in 100% of samples with the highest mean concentration at 376.79 ng/g fat, followed by endosulfan (70.32 ng/g fat) and β-HCH (65.32 ng/g fat). Elevated OCP levels were especially noted in Bekaa, Baalbek-Hermel, and South and North Lebanon, likely reflecting intensive agricultural practices and the ongoing use of contaminants. Estimated daily intakes (EDIs) indicated potential dietary exposure risks, particularly from PCBs, ΣDDTs, and ΣHCH. These findings underscore the urgent need for regulatory oversight and national food safety monitoring to ensure public health protection.

## 1. Introduction

Yogurt, a staple of the Mediterranean diet, is valued for its nutritional benefits and health-promoting properties, largely attributed to its content of bioactive peptides, prebiotics, and probiotics [1,2]. As a milk-derived product resulting from bacterial fermentation, yogurt is recognized as one of the most nutritious foods in the human diet, due to its easy digestibility and the high bioavailability of its vital nutrients [2]. These include macronutrients and micronutrients, notably proteins, essential minerals, and B-complex vitamins, which have contributed to the steady increase in its consumption across most countries over the past few decades [2,3]. Its widespread consumption across all age groups and its cultural value further underscores its dietary importance.

Emerging evidence highlights the health-promoting role of yogurt, notably in preventing several diseases, including metabolic, cardiovascular, and skeletal disorders, and in promoting digestive health and immune function [4,5,6]. Despite the above-mentioned beneficial health effects, concerns remain regarding the potential transfer of long-lasting environmental contaminants, mainly OCPs and PCBs, through the food chain via animal-derived products such as yogurt [7]. Given their lipophilicity, these compounds are prone to accumulating in fat-rich matrices, increasing the risk of contamination in dairy products [8,9,10,11,12]. Food consumption is recognized as the main non-occupational route of exposure to POPs, accounting for over 90% of total human intake [13].

In Lebanon, prior studies have confirmed environmental contamination, particularly in sediments, soil, and water sources, as well as in human exposure to POPs [14,15,16,17]. Locally, the Ministry of Environment is responsible for environmental regulation. However, the most recent policy issued by the Ministry in 2018 on solid waste management has not been effectively implemented, while environmental studies are typically conducted on a project basis by academic institutions with limited state involvement [18]. This lack of regular surveillance and integrated environmental data severely limits the country’s capacity to detect, prevent, or respond to contamination events, including those involving PCBs and OCPs, especially in widely consumed foods such as yogurt [19]. The absence of consistent monitoring is particularly concerning given the well-documented persistence of these compounds in the environment, their resistance to degradation, and their ability to bioaccumulate in animal and human tissues [20,21,22,23].

Among these contaminants, PCBs are of significant concern and may be classified into two major groups: non-dioxin-acting PCBs and dioxin-like PCBs [20]. The latter group, which includes coplanar and mono-ortho-substituted congeners, shares structural similarities and toxicological mechanisms of action with polychlorinated dibenzo-p-dioxins. In contrast, dioxin-like PCBs possess planar configuration and operate through different action mechanisms [24]. The majority of PCBs, 197 out of 209 congeners, are non-dioxin-like. The Stockholm Convention (SC) on POPs recommends monitoring six PCB congeners (PCB 28, 52, 101, 138, 153, and 180) to evaluate PCB pollution levels [20]. These six congeners are typically most abundantly present in each of the two matrices: the environment and human fluids [25]. Originally used as insulating fluids in electrical equipment, PCB emissions to the environment mainly originate from industrial sources [26], as well as from improper disposal, landfill leakage, inadequate incineration, and volatilization [26,27].

Despite Lebanon’s ratification of the Stockholm Convention in 2003 [28] and efforts to phase out PCBs by 2025, the major pollution sites are “Zouk” and “Jiyeh” electricity generation stations and “Bauchrieh” depot and workshop [29].

Similarly, OCPs are widespread toxic environmental pollutants extensively used worldwide [30]. This is particularly concerning as they raise international issues given their potential to cause harmful health implications and disrupt ecosystems [17]. Exposure to OCPs in humans occurs through cutaneous contact, inhalation, and primarily through the dietary consumption of contaminated foodstuffs, particularly lipid-rich items such as milk, dairy products, and fish, where these fat-soluble compounds tend to accumulate [30,31,32,33,34,35]. Moreover, OCPs readily evaporate and may be emitted into the environment via agricultural activities, household use, disposal of contaminated waste in both controlled and uncontrolled landfills, and emissions from stockpiles or production plants [36].

Several studies have shown that contact with PCBs and OCPs has been connected to cancerous, neurological, endocrine, and immune-related health impacts [37,38,39,40].

While some regional efforts have monitored milk and dairy products [41,42], and European countries have reported reassuring exposure levels [12,43], Lebanon still lacks systematic surveillance and monitoring programs for these contaminants in food [44]. This gap is particularly concerning for yogurt, which plays a central role in the Lebanese diet [1]. In fact, food safety oversight in the country is shared among several governmental bodies. Specifically, the Lebanese Food Safety Commission (LFSC), established under the Lebanese Food Safety Law No. 35/2015 [45], was created to centralize and coordinate food safety policies, inspections, and enforcement across the entire food chain. The law promotes a “farm to fork” strategy aimed at ensuring traceability, safety, and sustainability. However, effective implementation remains a challenge due to fragmentation of responsibilities and limited enforcement capacity [17,44]. These shortcomings are compounded by Lebanon’s pressing environmental and agricultural challenges, namely, unregulated farming practices, pollution, and weak adherence to international safety standards [19,44], all of which increase the likelihood of bioaccumulation in dairy matrices [15,16]. Additionally, the dairy industry itself contributes to environmental degradation due to its high energy demands along the production line and supply chain [46], and its intensive water usage, primarily for cleaning operations and equipment maintenance [47]. This results in the generation of significant volumes of wastewater, often containing elevated levels of organic and inorganic pollutants [48].

In this context, the present study is particularly timely. To the best of our knowledge, it is the first nationwide study to comprehensively evaluate both PCBs and OCPs in yogurt samples collected from multiple districts. Thus, by addressing this analytical and geographic gap, this study not only represents the first national-level dataset of its kind but also tackles a critical public health blind spot. It offers evidence-based insights to support national food safety policies, enhance risk assessment frameworks, and fulfill international obligations under the Stockholm Convention [28].

**Figure 1:**
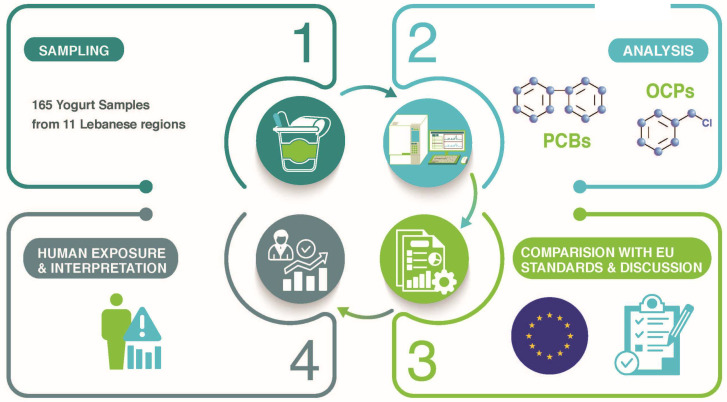
Study methodology scheme.

The objective of this article is to investigate the presence of six PCB congeners (PCB 28, 52, 101, 138, 153, and 180) and a range of OCPs, including (hexachlorobenzene (HCB), alpha-hexachlorocyclohexane (α-HCH), beta-hexachlorocyclohexane (β-HCH), gamma-hexachlorocyclohexane or Lindane (γ-HCH), Dichlorodiphenyltrichloroethane (DDT), Dichlorodiphenyldichloroethylene (DDE), Dichlorodiphenyldichloroethane (DDD), endosulfan, methoxychlor, and dieldrin in yogurt samples from across Lebanese regions, and to evaluate their potential contribution to dietary exposure. The analysis was conducted using gas chromatography. The research is designed to tackle a major gap in national food safety surveillance and provides critical insight into the contamination risks associated with dairy consumption in Lebanon, a country currently grappling with major challenges and declining progress toward SDG 2, which aims to eradicate hunger, ensure food security, improve nutrition, and promote sustainable agriculture. An overview of the study’s methodological workflow is presented in Figure 1, outlining key steps including sample collection and preparation, contaminant quantification using GC/ECD and GC/MS, and comparison with international safety standards, primarily the European Union (EU) Commission Regulation limits, in the absence of nationally adopted thresholds for PCBs and OCPs contamination. This is followed by exposure and risk assessment, and concludes with result interpretation.

## 2. Materials and Methods

### 2.1. Sampling

Yogurt samples were gathered from eleven distinct areas in Lebanon, namely Nabatiyeh (Na), South Area (S), Chouf (C), Metn (M), Keserwan (K), Jbeil (J), Batroun (B), North Area (No), Akkar (A), Bekaa (Be), and Baalbek-Hermel (BH). A total of fifteen samples were gathered from various dairy farms and small-scale processing facilities within each region, culminating in an overall sample size of 165. All yogurt products were prepared using artisanal-style methods within the farms’ facilities, with milk supplied from the same area. The production process used traditional methods, omitting the addition of any preservatives, stabilizers, or flavoring agents. The regional diversification of dairy production practices is showcased in these naturally made yogurts, which are widely consumed in Lebanon.

The acquired samples were promptly transported to the laboratory’s facility under refrigerated and sterile conditions. To prevent any degradation in pollutants levels or contamination of the samples, they were immediately analyzed. Additional portions were deep-frozen for later replication and confirmation.

**Figure 2:**
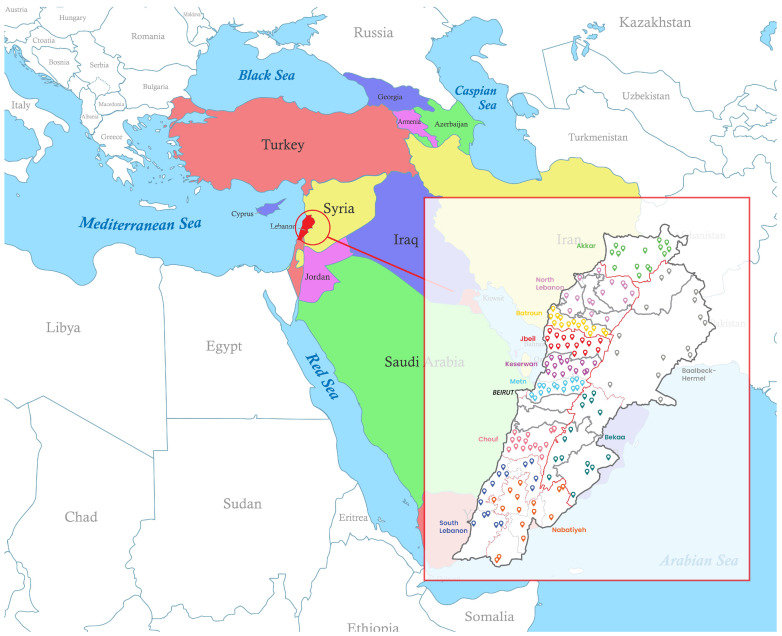
Map showing Lebanon’s position globally and the geographic spread of yogurt sampling sites across its eleven regions.

Figure 2 shows the location of the study regions in Lebanon and its location on the global map.

### 2.2. Extraction and Instrumental Analysis of PCBs and OCPs in Yogurt Samples

This study measured six PCB congeners: PCB 28, 52, 101, 138, 153, and 180; and ten OCPs: hexachlorobenzene (HCB), α-hexachlorocyclohexane (α-HCH), β-hexachlorocyclohexane (β-HCH), γ-hexachlorocyclohexane (γ-HCH), DDT, DDE, DDD, Endosulfan, Methoxychlor, and Dieldrin.

#### 2.2.1. Reagents and Materials

The analytical standards for PCBs and a standard mix of OCPs were acquired from Sigma Aldrich (Darmstadt, Germany). All solvents used in the process including acetonitrile, hexane, dichloromethane, acetone, and ethanol are of HPLC grade and sourced from Scharlau Chemicals (Barcelona, Spain).

#### 2.2.2. Sample Extraction and Cleanup

In this study, the extraction and purification of PCBs from yogurt samples were performed using a method analogous to that presented by Harmouche et al. [20]. Briefly, 10 g of uniformed yogurt was subjected to solvent extraction, during which an internal standard mixture (PCB 103 and PCB 193) was added to monitor recovery. The samples underwent sonication to facilitate the release of analytes, and then were processed using solid-phase extraction (SPE) for clean-up. The SPE cartridges were pre-conditioned with dichloromethane and a methanol–water solution, after which the extracts were loaded at a controlled flow rate. Following drying under a nitrogen stream and centrifugation, the analytes were extracted using a combination of hexane and dichloromethane. The eluates were then concentrated under gentle nitrogen evaporation at ≤40 °C. Further purification was performed through sequential cleanup steps over an aluminum sulfite (Al_2_O_3_) column, fractioned using a 1.5% (*w*/*w*) deactivated silica column followed by purification on an acidic silica column (40% H_2_SO_4_-silica).

OCPs were analyzed following the QuEChERS method outlined by Anastassiades et al. [49]. A 10 g portion of the homogenized sample was subjected to extraction using 10 mL of acetonitrile, followed by the addition of 4 g anhydrous magnesium sulfate (MgSO_4_) and 1 g sodium chloride (NaCl) to promote phase separation. The mixture was promptly agitated for 1 min and centrifuged to separate the layers; a 1 mL aliquot of the supernatant was cleaned using dispersive solid-phase extraction (d-SPE) by mixing with 150 mg anhydrous MgSO_4_ and 25 mg PSA sorbent, effectively removing polar interferences such as organic acids and pigments.

#### 2.2.3. Instrumental Analysis

Gas chromatographic analyses were conducted using an Agilent Technologies GC system (Agilent Technologies, Santa Clara, CA, USA), following a protocol similar to that mentioned by Harmouche et al. [18]. For the analysis of PCBs, the instrument featured a split/splitless injector alongside an electron capture detector (ECD), while OCPs were analyzed using a mass spectrometry (MS) detector. Chromatographic separation of PCBs was performed on a CP-8753 capillary column (60 m × 0.25 mm i.d., 0.25 μm film thickness; Agilent Chrompack, Santa Clara, CA, USA), with helium as the carrier gas flowing steadily at a rate of 1 mL/min. The oven temperature was programmed to hold at 90 °C for 3 min, ramp to 200 °C at 30 °C/min and hold for 15 min, followed by a ramp to 265 °C at 5 °C/min (5 min hold), and a final increase to 270 °C at 3 °C/min with a 15 min hold. For OCPs, separation was conducted using a CP-SIL 8 CB column (35 MS; 30 m × 0.25 mm i.d., 0.25 μm film thickness; Agilent Chrompack) with hydrogen as the carrier gas at 1 mL/min. The temperature program began at 110 °C (2 min hold), increased at 15 °C/min to 285 °C (5 min hold), and continued at 5 °C/min to 300 °C for a final 15 min hold. A 1 μL sample volume was injected in splitless mode for both PCB and OCP analyses.

Analytes were determined by matching their retention times and detector responses to those of certified external standards. Analytical standards were used to prepare calibration curves for quantification.

### 2.3. Quality Control and Assurance

Method blanks (1% KCl) were employed as part of the quality assurance process, along with internal standards (^13^C-labeled PCBs and surrogate OCPs) and certified reference materials. The mean recovery rate for PCBs was 84%, while for OCPs, recoveries varied between 88% and 94% depending on the compound. Limits of detection (LODs) were calculated based on 3.3 times the standard deviation of the smallest detectable amount with a signal-to-noise ratio ≥ 3. Limits of quantification (LOQs) were estimated as three times the LOD values. The LODs, LOQs, and compound-specific recovery rates are provided in Table 1.

### 2.4. Daily Dietary Exposure and Risk Characterization

The estimated dietary intake (EDI, expressed in ng/kg body weight/day) of pesticide compounds and the combined six PCBs in the yogurt was calculated using Equation (1):

EDI = C × MS/b.w.,(1)

where C represents the mean concentration of pesticides or PCBs (ng/g, wet weight), MS is the daily yogurt consumption in grams, and b.w. denotes the body weight (kg) of adult consumers. The EDI was determined only for pesticide and PCB compounds detected above the limit of quantification (LOQ) in more than one sample. In this study, no values were found between the LOD and LOQ, with all quantifiable data above the LOQ, and thus suitable for inclusion in the EDI calculation. As for non-detected (ND) values, below the LOD, they were substituted with LOD/2 for the purpose of statistical analysis. According to Hoteit et al. [50], the average daily yogurt consumption among adults in Lebanon is 72.33 g, while the mean body weight of adult consumers is 73.85 kg [50]. Since data on average yogurt consumption for children and adolescents in Lebanon are currently unavailable, the EDI calculations were restricted to Lebanese adults only.

The chronic risk associated with the pesticides and six PCBs detected in milk was evaluated by calculating the hazard quotient (HQ) using Equation (2):

HQ = EDI/HBGV,(2)

where HBGV refers to the health-based guidance value, which includes toxicological reference values such as acceptable daily intake (ADI), tolerable daily intake (TDI), or reference dose. These values, used for chronic risk assessment, are established by the Joint FAO/WHO Meeting on Pesticide Residues [51], the United States Environmental Protection Agency [52], and the European Commission [53]. An HQ value less than 1 suggests no expected adverse effects on consumer health, whereas an HQ exceeding 1 indicates a potential risk of significant chronic health impacts.

### 2.5. Data Analysis

The data analysis was conducted using IBM SPSS Statistics version 26. Descriptive statistics, including the mean and standard deviation, were applied to summarize and compare the concentrations of PCBs and OCPs detected in yogurt samples from various Lebanese regions. To evaluate compliance with regulatory standards, one-sample two-tailed t-tests were employed to evaluate the observed contaminant levels against established maximum residue limits (MRLs), along with calculating the percentage of samples exceeding the MRLs across regions.

## 3. Results and Discussions

### 3.1. PCBs

The current study investigated the levels of six indicator PCB congeners (PCB 28, 52, 101, 138, 153, and 180) in yogurt samples collected from 11 Lebanese regions. The results presented in Table 2 indicate that 40.6% of the tested samples recorded total PCB concentrations exceeding the European Union (EU) maximum residue limit (MRL) of 40 ng/g of fat [54]. The percentage of individual samples exceeding the MRL within each region ranged from 13.3% (Metn) to 66.7% in Keserwan and 73.3% in South Lebanon, identifying the latter as potential contamination hotspots and demonstrating significant regional variation. Complementing these findings, Table 3 shows that 59.4% of all samples analyzed had non-detected PCB residues, further emphasizing the variability in contamination levels across regions. Notably, regions such as Metn (86.7%) and Jbeil (80.0%) recorded the highest percentages of non-detected values, indicating relatively lower exposure risk in these areas. In contrast, South Lebanon (26.7%) and Keserwan (33.3%) had the lowest percentages, indicating a higher potential exposure risk.

As presented in Table 4, the national average of total PCBs was 39.68 ng/g of fat, which is just below the MRL, indicating proximity to the regulatory threshold. However, substantial regional disparities were also observed, reinforcing concerns about the potential health implications for consumers, particularly in high-risk areas.

Among the surveyed regions, Keserwan exhibited the highest mean concentration, reaching 48.15 ng/g fat, followed by South Lebanon (45.91 ng/g fat), North Area (43.56 ng/g fat), Chouf (42.18 ng/g fat), and Baalbek-Hermel (40.58 ng/g fat). These values all surpass the MRL, indicating potential health hazards from prolonged exposure in these areas.

In contrast, other regions such as Metn (32.52 ng/g fat), Batroun (34.19 ng/g fat), Jbeil (35.60 ng/g fat), and Nabatiyeh (37.33 ng/g fat) displayed comparatively lower PCB concentrations, which were all below the regulatory threshold. The variation in total PCB levels is mirrored in the distribution of individual congeners, with PCB153 and PCB180 consistently showing the highest concentrations across most regions, contributing substantially to the total PCB burden.

**Figure 3:**
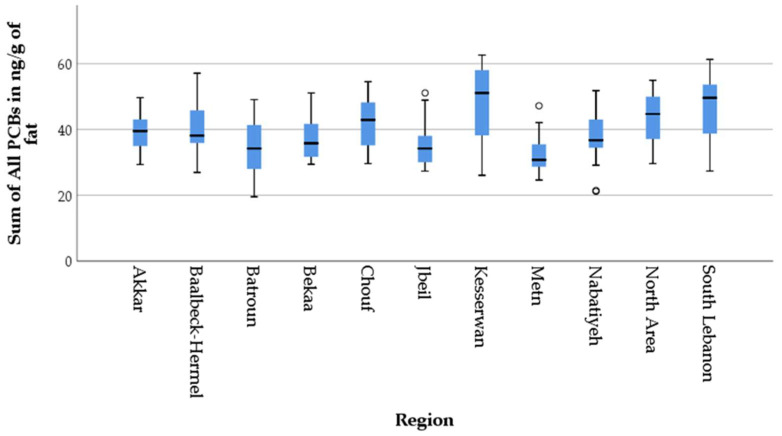
Box Plots of the Sum of All PCB_S_ in yogurt samples from the various Lebanese regions.

These quantitative findings are further supported by the box plot presented in Figure 3, confirming the numerical findings reported earlier and highlighting the regional disparities in the distribution of total PCBs across the studied regions, with several key insights. In the box plot, the circles represent outlying values, which are the observations that lie far from the mid-range of each dataset.

Notably, Keserwan, South Lebanon, North Area, Chouf, and Baalbek-Hermel present with higher medians and broader interquartile ranges, consistent with their mean values exceeding the EU MRL of 40 ng/g of fat. Keserwan shows the widest spread and highest upper whisker, suggesting not only a high median concentration but also a greater variability in contamination within the region. This may indicate heterogeneous exposure sources or varying degrees of environmental persistence and uptake.

South Lebanon and the North Area also display elevated median values and wide ranges, with some values extending above 60 ng/g. Chouf and Baalbek-Hermel similarly show central tendencies above the regulatory limit. In contrast, Metn, Batroun, and Jbeil show lower medians, narrower interquartile ranges, and a more compact distribution, suggesting more uniform and relatively lower contamination levels. Metn, in particular, has the lowest median and contains several outliers, indicating occasional high concentrations in an otherwise low-contamination context. Nabatiyeh also shows modest central values with a notable outlier below 20 ng/g, suggesting localized variation.

According to Cardellicchio et al. [26], emissions of PCBs into the environment primarily stem from industrial activities, along with improper waste disposal, leakage from landfills, incomplete incineration, and volatilization [26,27].

At the local level, Khalil Helou et al. [17] reported that transformer oils, formerly used by “Electricité du Liban” (EDL), which provide approximately 90% of Lebanon’s electricity, are the primary cause of PCBs pollution in the country. All EDL-managed facilities, including electricity generating sites, distribution stations, and workshops, are recognized as PCB hotspots. These sites encompass a total of 22,551 transformers containing transformer oils distributed across 7 thermal electricity generating stations, 12 hydropower stations, and 58 distribution stations nationwide. According to Shabani [55], the 7 thermal power plants using oil combustion methods are distributed in the Lebanese territory, as shown in Table 5.

As shown in Table 4, the major power plants in Lebanon are located in regions exhibiting the highest levels of PCB contamination in yogurt samples. Among the seven thermal power plants operated by EDL, the Zouk Power Plant, located in Keserwan, is the largest, with an output of 1003 MW. This makes it the most powerful oil-combustion facility in Lebanon.

This is particularly relevant given that Keserwan exhibited the highest total PCB concentration mean (48.15 ng/g fat) among all surveyed regions, well above the EU maximum residue level of 40 ng/g fat. The correlation between the high contamination level and the presence of the largest thermal power plant in the country strongly supports the hypothesis that the Zouk facility is a major contributor to regional PCB emissions.

This association is echoed in other regions as well. For example, South Lebanon, which reported the second-highest PCB level (45.91 ng/g), hosts two power plants: Zahrani (469 MW) and Tyr (70 MW), further confirming the relationship between thermal power generation capacity and environmental PCB load. Similarly, North Area, with a total PCB level of 43.56 ng/g, contains both the Deir Ammar (464 MW) and Hreisheh (75 MW) plants.

The Chouf region, which recorded a total PCB concentration of 42.18 ng/g of fat can be reasonably linked to its proximity to the Jieh power plant, a thermal oil-combustion facility with an output of 622 MW operated by Électricité du Liban (EDL). The Jieh plant is geographically situated along the coast within the administrative boundaries of Mount Lebanon, and in close vicinity to the Chouf district. Baalbek-Hermel also follows this trend, hosting a thermal facility with an output of 70 MW.

These findings reinforce the strong connection between power plant operation, especially facilities using oil combustion with high megawatt output, and elevated PCB concentrations in surrounding areas.

Although the Lebanese Parliament enacted Law 78 in 2018 on the air quality protection [56], which mandates the establishment of a national ambient air quality monitoring program, emission inventories, and threshold limit values, these provisions have yet to be fully implemented or operationalized in practice. In parallel, approximately 95% of the electricity in Lebanon is generated using fuel-oil in thermal power plants [57], a practice that significantly contributes to environmental emissions, including greenhouse gases (GHGs) and other harmful pollutants [58]. This heavy reliance on fuel-oil and gas-oil, coupled with the widespread use of diesel generators and the burning of low-quality fuels for residential and industrial heating, results in the release of persistent organic pollutants (POPs) into the environment. Despite these risks, Lebanon currently lacks binding air quality standards and does not mandate the use of filtration systems for private generators or industrial combustion sources, further exacerbating air pollution and posing serious threats to public health.

In a joint report performed by the UNDP and the Lebanese Ministry of Environment [59], PCB leakage was reported from improper storage or disposal of electrical equipment or transformer oils, especially in unregulated landfills, which can contaminate nearby soil and water sources affecting local feed and eventually milk supply.

In addition, other environmental sources may also contribute to the regional variability in PCB levels observed in yogurt samples. A study conducted in Tripoli Harbor, northern Lebanon, reported elevated concentrations of 28 PCB congeners across 15 coastal monitoring stations, particularly near port facilities and industrial zones [60]. These findings suggest that industrial and maritime activities contribute significantly to localized environmental PCB burdens. Such contamination can enter the agricultural chain through multiple pathways, including groundwater used for livestock and irrigation, soil deposition, and atmospheric transport of pollutants, serving as an indirect conduit for PCBs entering the dairy production chain.

On the other hand, the total levels of PCBs remained the lowest in the Batroun and Metn regions, suggesting possibly lower environmental emissions or agricultural contamination in those zones.

Studies investigating PCBs in Lebanon remain very limited, with most focusing on human biomonitoring rather than environmental contamination. For instance, although Harmouche et al. [20] reported that dairy products were not a significant source of PCB exposure in their urban human biomonitoring study, our findings provide complementary evidence from a food surveillance perspective; by directly measuring PCB levels in dairy fat across multiple regions, we identified significant environmental contamination, particularly in areas near thermal power plants. This suggests that regional environmental factors, rather than consumption habits alone, may drive PCB presence in dairy products, thereby reinforcing the role of local pollution sources previously acknowledged by Harmouche et al. [20].

Moreover, we have noticed considerable variation in the concentrations of individual PCB congeners across the surveyed regions. For example, PCB28 ranged from 1.679 ng/g of fat in Metn to 3.085 ng/g in Keserwan, while PCB153 ranged from 8.613 ng/g in Batroun to 12.987 ng/g in Keserwan, and PCB180 ranged from 9.900 ng/g in Metn to 14.160 ng/g in Keserwan. Notably, the most frequently detected and abundant congeners across all regions were PCB138, PCB153, and PCB180, which are higher-chlorinated PCBs containing five or more chlorine atoms. These congeners exhibited the highest mean concentrations, measured at 7.502 ng/g for PCB138, 10.024 ng/g for PCB153, and 11.685 ng/g for PCB180 at the national level (Table 6).

According to Table 6, the sum of these three high-chlorinated congeners ranged from 25.62 ng/g of fat in Batroun to 35.08 ng/g in Keserwan, while the sum of lower-chlorinated congeners PCB28, PCB52, and PCB101 ranged between 7.92 ng/g in Metn and 13.08 ng/g in Keserwan. On average, the higher-chlorinated PCBs accounted for approximately 74% of the total PCB burden, with PCB180 alone contributing 29.5%, which was the highest proportion among all congeners.

These results are consistent with the congener distribution patterns reported in Harmouche-Karaki et al. [14], who analyzed PCBs in serum samples of Lebanese adults and found that PCB138, PCB153, and PCB180 accounted for approximately 75% of the total PCBs, with PCB180 alone representing 34.1%. This predominance is in line with the well-documented resistance of these congeners to metabolic degradation. Indeed, PCB138, PCB153, and PCB180 are known to be poorly metabolized compared to lower-chlorinated congeners, which are more susceptible to enzymatic breakdown [61,62].

The recurring dominance of these congeners in both dairy and human biological matrices reflect their chemical stability, high lipophilicity, and low metabolic clearance rates. According to the ATSDR, these high-chlorinated PCBs can persist in human adipose tissue for periods ranging from 10 to 47 years, which explains their significant bioaccumulation in lipid-rich compartments such as dairy products, while low-chlorinated PCBs exhibit much shorter biological half-lives, which can reach up to 5 years [17].

It is important to highlight that, to date, no published data are available on the concentration of PCBs in Lebanese yogurt, and only a limited number of studies have assessed PCB levels in yogurt and other dairy products globally. Table 7 presents a comparison of non-dioxin-like PCBs (NDL-PCBs) concentrations in milk and yogurt reported in international studies, alongside the findings of the present research. Due to the scarcity of existing studies specifically on yogurt, milk was selected as an additional comparative matrix. This choice is scientifically justified, as yogurt is directly derived from milk, and both share comparable lipid content and contamination pathways, particularly for lipophilic compounds such as PCBs. Therefore, milk serves as a relevant reference matrix to support the evaluation of dietary exposure to PCBs through dairy consumption.

The comparison of our findings with international studies reveals that the mean concentration of PCBs in Lebanese yogurt samples (39.68 ng/g fat) is among the highest levels reported globally, surpassed only by data from Turkey [67] (127.27 ng/g fat) and significantly exceeding values observed in most European and North American countries. For instance, studies conducted in Canada and Denmark [10] reported mean concentrations below 2 ng/g fat, while Belgium [65] and Romania [66] recorded intermediate levels of 4.72 and 9.12 ng/g fat, respectively. Even in countries with intensive agricultural and industrial activity, such as Spain and Iran [12,69], average levels remained substantially lower than those found in Lebanon, with concentrations ranging from 12.55 to 14.65 ng/g fat.

The observed discrepancy highlights a critical gap in environmental and food safety governance. While many countries have implemented effective bans, remediation strategies, and routine surveillance for PCBs, Lebanon continues to face regulatory delays and insufficient enforcement mechanisms. According to Helou et al. [17], although Lebanon never produced PCBs, widespread historical use of PCB-containing insulating oils in thermal power plants, substations, and transformers operated by EDL has left a lasting legacy of environmental contamination. These infrastructures, distributed across the country, remain major hotspots for PCB release into surrounding ecosystems.

Moreover, the artisanal nature of local dairy production, often lacking formal quality control or standardized feed and water monitoring, may further contribute to contamination through environmental exposure or contact with polluted surfaces. These compounding factors, such as persistent pollution, weak regulatory oversight, and informal food processing practices, likely explain Lebanon’s elevated PCB levels in dairy products when compared to countries with stronger environmental policies and industrial controls.

In light of these concerns, the Ministry of Environment should enhance its oversight of food production activities and promote best environmental practices. Food production facilities, particularly milk processing industries known for their high energy consumption and wastewater generation, must also be pressured to adopt energy-efficient technologies, install proper air filtration systems, and ensure the treatment of wastewater generated during processing. Such measures are crucial for enhancing environmental performance and promoting sustainable practices in food production.

### 3.2. OCPs

The results of OCP analysis in all yogurt samples collected from the 11 Lebanese regions revealed widespread contamination across all tested OCP compounds, with statistically significant differences observed among regions (*p* < 0.001) for most pesticides. The mean concentrations of all OCP compounds in all regions were significantly higher than their corresponding maximum residue limits (MRLs) defined by the Commission Regulation (EU) 299/2008 [70], suggesting a potential public health concern from dietary exposure to these persistent pollutants. At the regional level, 100% of the samples exceeded the MRL for ΣDDTs, while high exceedance rates were also recorded for α-HCH (95.1%), γ-HCH (89.1%), β-HCH (87.9%), dieldrin (86.7%), methoxychlor (85.5%), HCB (84.9%), and endosulfan (77.6%) (Table 8). Regional analysis revealed that Bekaa, Baalbek-Hermel, and South Lebanon had the highest frequencies of exceedance for multiple OCPs, pointing to localized sources or agricultural practices contributing to elevated exposure risk. Complementing these findings, Table 9 shows low non-detection rates for all OCPs, confirming the widespread contamination of yogurt samples. No samples were free of ΣDDTs, and for most compounds, non-detected residues were found in less than 30% of samples. South Lebanon had the highest percentage of samples with non-detected OCPs, with four out of eight compounds showing absence of non-detected results. Similarly, Bekaa and Baalbek-Hermel showed very low non-detection rates across most OCPs. In contrast, Metn recorded the highest non-detection percentages for several compounds, including endosulfan (53.3%), suggesting relatively lower contamination in that region. North Area stood out with a high non-detection rate for endosulfan (96.7%), though detection remained high for other pesticides. These patterns highlight significant regional disparities, likely linked to local agricultural practices.

Specifically, mean concentrations, reported in Table 10 and Table 11, across regions were found to be higher than the MRLs of 10 μg/kg for each HCB, α-HCH, β-HCH, and γ-HCH; 50 μg/kg for endosulfan; 10 μg/kg for methoxychlor; and 6 μg/kg for dieldrin. In addition, the levels of organochlorine degradation products, DDT, DDE, and DDD, were markedly elevated across all regions, with total DDT-related compounds (ΣDDTs) far surpassing the MRL of 40 μg/kg fat, further underscoring the contamination burden [67].

As shown in Table 10 and Table 11, regional variation in OCP concentrations is noticed, reflecting differences in agricultural activities and environmental contamination. Regional comparison highlighted that yogurt samples from the Bekaa region exhibited the highest levels of OCPs, followed by Baalbek-Hermel, and the South and North areas. These areas are known for their intensive agricultural practices, which contribute to persistent pesticide residues through soil and water contamination. This aligns with Darwish et al. [71] who reported that the Central Bekaa Plain, including Baalbek-Hermel governorate, represents Lebanon’s primary region for high-quality farmland. However, intensive agricultural practices, urban development, and industrial activities are putting increasing strain on the area’s already limited soil and water resources [72].

Moreover, a study performed by El Osmani et al. [73] has shown that the northern area in Lebanon, which ranks as the second most agriculturally active region in the country, suffers from extensive pesticide misuse given the excessive agricultural activity. The tested groundwater samples were highly contaminated with OCPs, far exceeding Stockholm Convention limits [73]. The contamination was particularly pronounced during planting seasons, suggesting seasonal spikes due to intensified pesticide use [73]. This groundwater is commonly used for irrigation and livestock drinking, creating a pathway for OCPs to enter the food chain through contaminated forage and water consumed by dairy cattle.

Similarly, water pollution by OCPs has been documented in other major agricultural zones, including the South Litani River Basin located in the South Lebanon area [72]. Youssef et al. [72] reported significant levels of DDE in both surface and groundwater used for agriculture and drinking. These findings underline the persistence of OCPs in the Lebanese water systems and their potential to enter the food chain through contaminated irrigation and livestock drinking water. As dairy cattle consume water and feed from such environments, these contaminants may accumulate in milk fat and eventually appear in yogurt.

These findings align with the national monitoring data presented in the Helou et al. study [17], stating that agricultural lands constitute primary sites of OCP contamination, such as the Bekaa Plain, North Area, and Akkar.

Lebanon does not manufacture pesticides and entirely ship in all its agricultural needs [27,28,72]. Although the Ministry of Agriculture maintains a registration list for banned pesticides and growth regulators [74], the country’s agricultural sector faces significant regulatory gaps, including weak market surveillance and inconsistent border control over unauthorized substances. As a result, banned OCPs are still smuggled into the country through official harbors and airports, in addition to uncontrolled segments of the northern and eastern frontiers shared with Syria [17].

Moreover, OCPs are often misused or mishandled by farmers, who also dispose their containers improperly, which additionally contributes to environmental contamination [17]. Hence, Lebanon currently lacks the capacity to effectively control both the illegal influx and the improper use of OCPs [75]. Furthermore, it is evaluated that less than 0.1% of applied pesticides access their target crops, while the majority end up contaminating the air, soil, and water [75]. Consequently, agricultural fields, along with nearby rivers and streams, are susceptible to being polluted. In Lebanon, gravity-based irrigation is the most common method, which facilitates the transport of OCPs from farmlands into surface and groundwater systems [17,75].

Additionally, several studies have documented high pesticide residues in soils across multiple villages, as well as in various vegetable crops such as lettuce, cabbage, tomatoes, and corn, often exceeding their respective MRLs [44,76]. This suggests a high likelihood of contamination throughout the broader agricultural environment, including livestock feed. Given that animals often consume locally grown forage and use irrigation water sourced from these areas, contaminated feed represents a critical exposure route. The resulting bioaccumulation in cattle can lead to detectable OCP residues in milk and yogurt, providing a supplementary plausible explanation for the regional contamination patterns observed in our study.

Comparatively, Metn, Batroun, and Jbeil showed relatively lower contamination levels, though still above MRLs, likely due to less agricultural activity and lower pesticide usage in these areas. Nevertheless, even these regions were not exempt from significant contamination, indicating a broader environmental issue affecting the national dairy supply chain.

This widespread environmental contamination explains the presence of OCP residues in the feed and drinking water supplied to dairy cattle. Consequently, these compounds can build up in living beings and increase in concentration up the food web, ultimately contaminating food sources, particularly those of animal origin, and potentially reaching human consumers [77,78].

**Figure 4:**
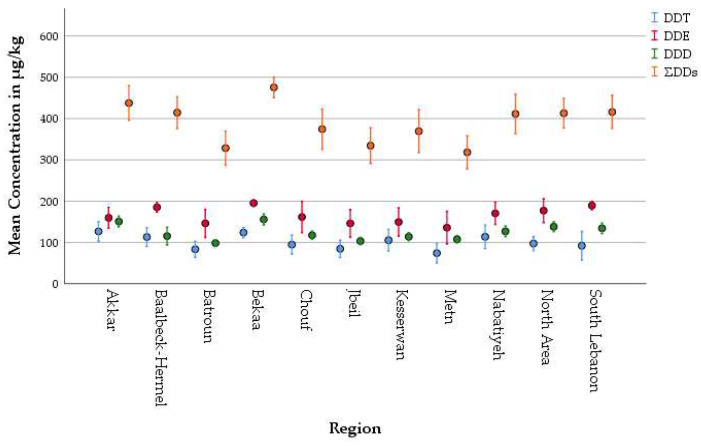
Simple error bar graphs of DDT compounds and their total concentrations in yogurt samples from various Lebanese regions.

**Figure 5:**
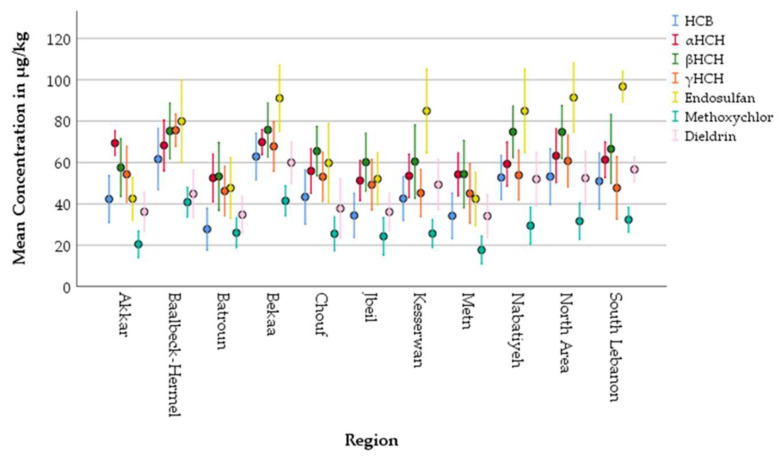
Simple error bar graphs of the OCP compounds in yogurt samples from the various Lebanese regions.

When examining individual compounds across regions, disparities can be observed and are clearly illustrated in Figure 4 and Figure 5. As shown in Figure 3, DDE, DDD, and DDT consistently showed peak levels, particularly in Bekaa (195.33 μg/kg, 155.93 μg/kg, and 124.13 μg/kg fat, respectively) and Akkar (159.76 μg/kg, 150.87 μg/kg, and 126.88 μg/kg fat, respectively). In Lebanon, DDT and its metabolites were banned since 20 May 1998 [74], and these results suggest both historical persistence and possible ongoing illegal use of DDT and its metabolites despite national bans. Notably the predominance of DDT metabolites followed this order, p,p′-DDE > p,p′-DDD > p,p′-DDT, which is aligned with the half-lives of DDT compounds measured in cows by Fries [79] who demonstrated that DDE remained persistent, while DDT was converted to DDD through rumen metabolism. This congener pattern is consistent with findings from Helou et al. [17], who indicated OCP contamination in Lebanese rivers, sediments, and underground water. Particularly, in the Hasbani River, which flows through southeastern Lebanon, DDE was identified as the most prevalent contaminant in three studies, with levels greatly surpassing the maximum permissible limits established by the EPA (8.3 ng/L) moreover its level in the groundwater Hasbani basin exceeded 100 ng/L, which aligns with the peak concentration of DDE found in the yogurt samples collected from the South Lebanon area (189.47 μg/kg fat) [17]. In addition, the sediment samples from the Kebir river in North Lebanon featured different concentrations of HCB, DDT, and DDE; the highest being DDT with an average sum of 3840 ng/g, aligning with our findings in Akkar (126.88 μg/kg fat), and exhibiting the highest concentration of DDT in the tested samples among other Lebanese regions [17]. The detection of DDT and its metabolite DDE suggests the long-term environmental persistence of this compound as well as continuous illegal application of this banned pesticide.

Similarly, Endosulfan and β-HCH were widely detected across the surveyed regions, with the highest concentrations observed in the Bekaa, South Lebanon, and Baalbek-Hermel regions (Figure 5), further highlighting the persistent use of legacy agrochemicals and the lack of effective regulatory oversight. Although Endosulfan was officially banned in Lebanon as of 13 February and 14 December 2010, and β-HCH since 20 May 1998 [71], their continued presence suggests ongoing illegal use and environmental persistence. Endosulfan concentrations ranged from 53.62 μg/kg of fat in Akkar to 96.73 μg/kg of fat in South Lebanon, with 82% of the regional mean values exceeding the European Union maximum residue level (MRL) of 50 μg/kg of fat. This distribution pattern likely reflects the intensive agricultural practices prevalent in these areas, where Endosulfan may have been historically or illicitly applied for pest control in both crop and animal feed production systems. As for β-HCH, its levels ranged from 53.62 μg/kg of fat in Batroun to 75.75 μg/kg of fat in Bekaa with 100% of mean concentrations exceeding the EU MRL (10 μg/kg fat). α-HCH and γ-HCH followed comparable regional patterns, with the highest values again in Bekaa and Baalbek-Hermel, suggesting either extensive legacy use or environmental persistence. Notably, γ-HCH, also known as Lindane, reached levels as high as 69.99 μg/kg fat in Bekaa, underscoring the widespread distribution of this pesticide.

As presented in Figure 4, HCB, Methoxychlor, and Dieldrin also exhibited a concerning regional distribution pattern across the analyzed yogurt samples. The highest levels for these compounds were generally concentrated in Bekaa, Baalbek-Hermel, and South and North Lebanon, regions characterized by intensive agriculture. HCB concentrations ranged from 27.75 μg/kg fat in Batroun to a peak of 62.89 μg/kg fat in Bekaa, far exceeding the European Union maximum residue level (MRL) of 10 μg/kg fat. Methoxychlor, although not individually banned in Lebanon, falls under the list of persistent organic pollutants (POPs) prohibited by the Convention of Stockholm, to which Lebanon is a signatory. Its highest concentration was observed in Bekaa (41.49 μg/kg fat). Dieldrin, banned since 20 May 1998, and β-HCH, banned since 24 December 2008 [74], were also detected at worrying levels. Dieldrin concentrations were particularly elevated in Bekaa (59.95 μg/kg fat), South Lebanon (56.67 μg/kg fat), and Nabatiyeh (51.97 μg/kg fat), far surpassing the MRL of 4 μg/kg fat. These findings highlight not only the lasting presence of these substances in the environment but also the likely continuation of illicit deployment and insufficient implementation of current pesticide policies.

Comparison with biomonitoring data from Helou et al. [16], which assessed OCP residues in human milk in Lebanon, aligns with the high concentrations of DDE and DDT found in our collected yogurt samples as the most prevalent OCPs in the milk samples were DDT residues (DDT, DDD, and DDE) with DDE detected in 97% of samples. The DDE concentrations in milk ranged from 47 mg/L to 1563 mg/L, and the mean DDE value was 362 ± 34 mg/L [17].

Similarly, in the Harmouche-Karaki et al. [14] study, OCPs were evaluated in Lebanese adults’ serum, and DDE was the highest quantifiable compound with a mean concentration value of 18.9 ng/g lipids.

In another study by Harmouche-Karaki et al. [20], assessing serum OCP levels in Lebanese adults, the findings support the hypothesis that food is the principal transmission route for OCPs. In their study, the consumption of animal-based food, including dairy products, is positively associated with a high level of serum OCP concentrations. This is particularly relevant given that yogurt, a fat-rich food, facilitates the bioaccumulation of lipophilic compounds such as DDT and DDE among other OCPs [20]. When cattle consume pesticide-contaminated crops, biomagnification occurs along the food chain, resulting in the presence of these chemicals in both meat and milk [80].

A comparative evaluation of organochlorine pesticide (OCP) residues in yogurt and milk products across several countries, presented in Table 12 and Table 13, highlights concerning discrepancies between international and Lebanese levels. In our Lebanese yogurt samples, OCP concentrations expressed on a fat basis were markedly elevated, with the highest concentrations for ΣDDTs, ΣHCH, and Endosulfan reaching 390.22 μg/kg, 179.66 μg/kg, and 70.32 μg/kg fat, respectively. These figures significantly exceed those reported internationally. For instance, in the Middle East, Nida’M et al. [41] conducted a study in Jordan assessing OCP residues in 233 dairy product samples, including butter, cheese, milk, labneh, and yogurt. Their findings showed that β-HCH, p,p′-DDE, α-HCH, and γ-HCH contaminated 9% (21/233), 8.5% (20/233), 6% (14/233), and 2.1% (5/233) of the samples, respectively [41]. Heptachlor and α-Endosulfan were present in less than 2% of the tested products [41]. Aldrin, o,p′-DDD, p,p′-DDD, o,p′-DDE, o,p′-DDT, p,p′-DDT, dieldrin, β-Endosulfan, endrin, and HCB were not detected in any of the samples, contrasting with our findings [41], where most tested OCP compounds exceeded their respective MRLs in the surveyed yogurt samples. Numerically, Jordanian yogurt and milk samples exhibited values notably lower than those reported in our study: ΣHCH and ΣDDT levels of 94 and 32 μg/kg fat in yogurt, respectively, while milk samples showed levels of 133 μg/kg fat for ΣHCH, 27 μg/kg fat for ΣDDT, and 30 μg/kg fat for Endosulfan [41]. Whereas research carried out by El Makarem et al. [42] in Egypt on raw bovine milk samples gathered from three distinct governorates detected the presence of DDT, dieldrin, endrin, and lindane in all samples, with mean concentrations exceeding their respective MRLs; these findings are consistent with the results obtained from our yogurt samples.

In Ethiopia, Ghebremichael et al. [81] and Deti et al. [82] reported ΣDDT concentrations ranging between 72.5 and 555 μg/kg fat in cow milk, reflecting significant variability due to geographic and temporal factors. In contrast, European levels were substantially lower. Rodríguez-Hernández et al. [12] reported ΣHCH, ΣDDT, and Dieldrin concentrations of 0.59, 7.63, and 7.98 μg/kg fat, respectively, in Spanish conventional yogurt, and 0.74, 5.65, and 0 μg/kg fat, respectively, in Spanish organic yogurt, with non-detectable levels of Endosulfan and Methoxychlor underscoring the effectiveness of regulatory controls in the European Union.

When the Lebanese data were presented on a fresh-weight basis to facilitate comparison with the wider literature, the concentrations remained among the highest reported worldwide, reaching 13.66 μg ΣDDT/kg, 6.29 μg ΣHCH/kg, and 2.46 μg endosulfan/kg. Over the past 15 years, Chinese monitoring studies have illustrated the benefits of stringent regulations: the levels of ΣHCH and ΣDDT in milk were 0.60 μg/kg and 0.50 μg/kg, respectively, in 2006 [83]; they increased slightly to 0.63 μg/kg and 2.29 μg/kg, respectively, in 2017 [78]; and then they declined to 0.07 μg/kg and 0.10 μg/kg, respectively, in 2021 [89]. A similar downward trend is evident in India, where concentrations fell from 162 μg ΣHCH/kg, 172.4 μg ΣDDT/kg, and 49.2 μg Endosulfan/kg in 2008 [84] to ranges of ND–1.24 μg ΣHCH/kg, 0.53–1.70 μg ΣDDT/kg, and ND–1.24 μg Endosulfan/kg in 2020 [87]. Polish surveys [85] reported intermediate levels of 1.72–3.75 μg ΣDDT/kg, and 0.72–4.03 μg ΣHCH/kg, while even lower residues were documented in other European countries: Romanian milk [88] contained only 0.1137–0.218 μg ΣDDT/kg, and Croatian milk [43] showed 1.04 μg ΣHCH/kg, 1.7 μg ΣDDT/kg, 1.68 μg Endosulfan/kg, 1.78 μg Methoxychlor/kg, and 1.1 μg Dieldrin/kg, underscoring the effectiveness of EU regulatory controls. By contrast, an Egyptian survey [86] reported markedly higher values of 48.65 μg ΣHCH/kg, 54.77 μg ΣDDT/kg, and 13.96 μg Dieldrin/kg, reflecting continued, largely unregulated use of banned pesticides.

This alarming contrast is further underscored by the environmental context in Lebanon. Although the country does not manufacture OCPs, continued illegal use, smuggling, and inadequate monitoring have led to widespread environmental contamination. This is intensified by artisanal dairy production methods and proximity to agriculturally intensive areas, which likely contribute to higher contamination levels in the dairy supply. These findings, which are in line with Lebanon’s performance on SDG 2, emphasize the urgent need for strengthened regulatory controls, national monitoring programs, and food safety interventions to mitigate exposure risks and align with international standards.

To achieve this, the implementation of a comprehensive “farm to fork” strategy is essential for strengthening traceability, safety, and sustainability across all stages of the dairy value chain, from raw milk production to final consumption. In Lebanon, where food safety oversight is fragmented across institutions [44], adopting such an integrated approach would improve coordination, reinforce risk assessment, and support compliance with Good Agricultural Practices (GAP), Good Manufacturing Practices (GMP), and Hazard Analysis and Critical Control Points (HACCP) standards. Moreover, it would operationalize the Lebanese Food Safety Law (No. 35/2015) [45] and help restore public trust in locally produced dairy products.

### 3.3. Dietary Exposure and Risk Assessment

Table 14 outlines the dietary exposure and possible long-term health risks linked to the consumption of artisanal yogurt in Lebanon, focusing on the analyzed PCB and OCP compounds. To calculate the EDI values, the average concentrations of the compounds were converted to a fresh weight basis using an average fat content of 3.5%, which represents the mean fat content of the yogurt samples analyzed in this study. The fat content across the sampled products ranged between 3.1% and 3.9%, depending on the milk source and production method. While this standardization facilitates comparison with previous studies, it may introduce minor variation in EDI estimations due to natural fat content variability.

The health-based guidance values (HBGVs) used in this assessment include reference doses (RfDs), tolerable daily intakes (TDIs), acceptable daily intakes (ADIs), and provisional tolerable daily intakes (PTDIs), depending on the compound. Specifically, the RfD for ΣPCBs and ΣHCH were set at 20 ng/kg bw/day and 2000 ng/kg bw/day, respectively [43], and the TDI for hexachlorobenzene (HCB) was 170 ng/kg bw/day as established by WHO/IPCS [90]. The values for ΣDDTs (10,000 ng/kg bw/day), endosulfan (6000 ng/kg bw/day), and dieldrin (100 ng/kg bw/day) were based on WHO/JMPR ADI and PTDI assessments [51], while the RfD for methoxychlor (5000 ng/kg bw/day) was derived from U.S. EPA guidance [91].

The estimated daily intake (EDI) of PCBs from Lebanese yogurt was 1.36 ng/kg body weight/day and is situated within the lower spectrum of EDI estimates reported in Europe according to EFSA [92], where average exposures range from 4.3 to 25.7 ng/kg bw/day for the sum of six non-dioxin-like PCBs (NDL-PCBs). Comparative data on dietary PCB exposure highlight variations across countries and food sources. A French study from 2012 [93] observed a low EDI value of 0.046 ng/kg bw/day, with a decline in PCB intake from meat, while dairy products remained a consistent source of exposure. In Austria [94], estimated PCB intakes ranged from 2.64 to 3.19 ng/kg bw/day for adults, with milk and dairy products accounting for 50–55% of total PCB exposure. Similarly, a study in Turkey [95] reported lower EDI values of 1.14 ng/kg bw/day for adults and 2.97 ng/kg bw/day for children from milk consumption. In Australia [96], Total Diet Study results showed EDI ranges of 0.015–2.3 ng/kg bw/day for adults and 0.0032–3.9 ng/kg bw/day for children aged 2–17 years. Compared to these findings, the Lebanese yogurt-based EDI of 1.36 ng/kg bw/day is within the international range, though it is noteworthy given that it stems from a single dairy product. As for the Hazard Quotient (HQ) for PCBs, although the absolute value is relatively low (0.068), it remains a relevant indicator of cumulative exposure, particularly in the context of multiple contaminated food items. Given that dairy products are among the primary contributors to PCB intake, this finding underscores the need for continued monitoring of Lebanese dairy products, especially those from artisanal sources.

For OCPs, EDI values varied between 0.98 and 13.44 ng/kg bw/day, with ΣDDTs exhibiting the highest intake (13.44 ng/kg bw/day), corresponding to 0.13% of the WHO/JMPR HBGV (10,000 ng/kg bw/day) [51], while methoxychlor is the lowest at 0.98 ng/kg bw/day; intermediate values include ΣHCH (6.16 ng/kg bw/day), endosulfan (2.41 ng/kg bw/day), dieldrin (1.59 ng/kg bw/day), and HCB (1.58 ng/kg bw/day). While all calculated HQs remained well below 1 (ranging from 0.0002 for methoxychlor to 0.0159 for dieldrin), the comparative exposure remains significant. For instance, total diet studies in Croatia [43] reported EDIs of 3.33 ng/kg bw/day for ΣDDTs, 3.30 for Σendosulfan, 2.04 for ΣHCH, 1.80 for HCB, and 3.49 for methoxychlor. Catalonia milk [97] exhibited an EDI value of 2.03 ng/kg bw/day HCB; Nanjing adults [83] received 29.13 ng/kg bw/day ΣDDTs and 12.62 ng/kg bw/day ΣHCH; Hong-Kong milk [98] contributed 23.8 ng/kg bw/day ΣDDTs and 8.5 ng/kg bw/day endosulfan; and Turkish milk [95] yielded 2.59 ng/kg bw/day ΣDDTs, 1.86 ng/kg bw/day Σendosulfan, 0.43–1.13 ng/kg bw/day methoxychlor, and only 4.78 ng/kg bw/day ΣHCH.

None of the individual HQs exceeded the threshold value of 1, indicating no immediate carcinogenic health risk from yogurt consumption. However, among the compounds assessed, ΣPCBs (HQ = 0.068) and dieldrin (HQ = 0.0159) demonstrated the highest relative risk indices, suggesting they are the most significant contributors to the overall health risk. Moreover, PCB-153 may also exhibit particular concern, as it was frequently reported as one of the most prominent congeners in various studies, indicating higher environmental persistence and suggesting its disproportionate role in total PCB burden [99,100,101]. Although ΣDDTs had the highest EDI, its HQ remained low due to a high ADI value.

While the present study exhibits non-carcinogenic risk, it is important to highlight that several compounds under investigation, particularly PCB mixtures and dieldrin, are recognized for their carcinogenic potential. According to the International Agency for Research on Cancer (IARC), PCBs are classified as Group 1 carcinogens (carcinogenic to humans) [102], while dieldrin is classified as Group 2A (probably carcinogenic to humans) [103]. In addition, the U.S. Environmental Protection Agency (EPA) has established oral cancer slope factors (CSFs) for these substances: 2.0 mg/kg/day^−1^ for PCBs [104] and 1.6 mg/kg/day^−1^ for dieldrin [105]. Although incremental lifetime cancer risk (ILCR) calculations were not conducted in this study, future assessments should incorporate carcinogenic risk on a wide range of food matrices to provide a more comprehensive evaluation of potential long-term public health impacts.

These findings suggest that, even when HQs do not exceed the safety threshold of 1, the consistently elevated EDIs across multiple OCPs in Lebanese yogurt signal a potential cumulative exposure concern. This is especially relevant when considering the lipophilic nature and bioaccumulative behavior of OCPs, which could contribute to chronic health hazards, notably among high-risk populations, including children, expectant women, and high consumers of dairy products. The data underline the urgent need for enhanced surveillance, strengthened enforcement of pesticide restrictions, and broader assessments of dietary exposure from locally produced animal-origin foods.

A key limitation of this risk assessment study is the absence of national consumption data for children and adolescents in Lebanon, which restricted dietary exposure and risk assessments to the adult population. Future research should aim to include younger age groups to better evaluate the risks for more vulnerable consumers.

## 4. Sustainability Considerations in PCB and OCP Contamination of Dairy Products

The sustainable management of persistent organic pollutants (POPs), including PCBs and OCPs, in dairy products, such as yogurt, is critical to protecting human health and maintaining food safety. The latter means that it does not align only with SDG 2 (Zero Hunger) but also supports several other SDGs, notably SDG 3 (Good Health and Well-being), SDG 6 (Clean Water and Sanitation), and SDG 12 (Responsible Consumption and Production) [106]. In the context of Lebanon’s dairy sector, these contaminants primarily originate from polluted animal feed, contaminated water sources, or packaging materials, making it vital to address their presence through holistic and sustainable approaches.

One viable solution is bioremediation, which can be applied to contaminated agricultural soils used for growing fodder. Microbial communities capable of degrading chlorinated compounds in the soil can indirectly reduce the accumulation of these pollutants in dairy supply chains. This low-energy, eco-friendly approach supports ecosystem recovery and aligns with SDG 3 through improved environmental health and SDG 15 (Life on Land) by promoting ecosystem regeneration [106]. Bioremediation also improves soil quality and reduces the need for synthetic agrochemicals, reinforcing SDG 12.

Another sustainable technique is the use of biochar, derived from agricultural waste, to adsorb PCBs and OCPs from animal drinking water or farm effluents before they reach the dairy animals. Biochar’s porous surface makes it effective in capturing hydrophobic contaminants, while its production follows circular economy principles by valorizing waste biomass [107]. This supports SDG 6 by ensuring cleaner water sources for livestock and contributes to SDG 13 (Climate Action) by promoting carbon sequestration.

In addition, phytoremediation can be employed in grazing lands or irrigation areas surrounding dairy farms. Plants with the ability to uptake or stabilize organic pollutants can prevent contamination from spreading through feed and water pathways into the food chain. It supports SDG 13 through passive, solar-driven pollutant mitigation and SDG 15 by preserving soil and biodiversity health, especially in regions like Lebanon where natural resources are limited and ecological preservation is crucial [108].

A more advanced option is synergistic green remediation, which combines the benefits of bioremediation, phytoremediation, and biochar. This method is particularly effective in complex agricultural landscapes and can reduce the risk of POP bioaccumulation in livestock and their dairy outputs. By improving soil fertility and microbial activity while intercepting pollutants, it directly targets SDG 2 by contributing to safer and more sustainable dairy production, ensuring both nutritional quality and food security, along with SDG 3, SDG 12, and SDG 15 [109].

A comparative summary of these remediation methods and their relevance to sustainable dairy production and yogurt safety is provided in Table 15.

These methods vary in applicability and effectiveness based on soil type, infrastructure, and scale. For example, biochar is ideal for decentralized farms using rainwater or well systems but may require filtration systems for high-organic load water [107]. Bioremediation is flexible but can take longer to show results, particularly in mixed-contaminant agricultural soils [106]. Phytoremediation works best in rural, non-urban areas where field conditions are manageable [107]. Finally, synergistic remediation can be adapted for larger farms or dairy cooperatives in Lebanon aiming to reduce contamination risk across the full milk supply chain [109].

## 5. Conclusions

This research presents the first extensive nationwide assessment of persistent organic pollutants (POPs), namely PCBs and OCPs, in 165 yogurt samples collected from farms across 11 Lebanese regions. The results revealed a concerning prevalence of banned contaminants in the majority of samples, with several compounds exceeding international safety thresholds. Particularly, the levels of ΣDDTs, ΣHCH, and Dieldrin were alarmingly high, approximately tenfold higher than their corresponding MRLs. Although the derived hazard quotient (HQ) values for individual OCPs remained below 1, the elevated EDIs raise concern for potential cumulative effects, especially in the context of chronic dietary exposure, potentially increasing the risk of adverse health effects, particularly in vulnerable populations. Regional disparities in contamination levels aligned with agricultural intensity, environmental proximity to pollution sources such as power plants, and known regulatory gaps in pesticide control. The detection of banned compounds like DDT, dieldrin, and endosulfan decades after their prohibition reflects both environmental persistence and ongoing illegal use.

These findings highlight the urgent need for a national response to address PCB and OCP risks in Lebanon. The absence of monitoring in food and environmental matrices, especially dairy, underscores the need for robust surveillance and clear regulatory frameworks monitored by effective governmental bodies. Public health protection requires institutional and legal reforms as well as improved agricultural and industrial regulation. Strengthening governmental oversight and expanding environmental monitoring are also essential. Targeted awareness campaigns can further support sustainable practices and risk reduction. Furthermore, the integration of sustainability principles into national food and environmental policies is vital, addressing not only chemical safety but also the long-term ecological consequences of persistent pollutants. Such efforts should align with broader Environmental, Social, and Governance (ESG) frameworks to promote a resilient and health-conscious governance model. Accordingly, further research is strongly recommended, including longitudinal biomonitoring studies, surveillance of major food categories, identification of contamination hotspots, and cumulative risk assessments, to support the development of a comprehensive set of measures for more effective risk management and regulatory oversight. In this context, studies that bridge scientific evidence with institutional and policy frameworks are crucial to strengthening governance and improving food safety systems in Lebanon.

## Figures and Tables

**Table 1:** LOD, LOQs, and recovery rates for the analyzed compounds.

Compound	LOD (μg/kg in Extract)	LOD (μg/kg Fat Basis)	LOQ (μg/kg in Extract)	LOQ (μg/kg Fat Basis)	Recovery (%)
PCBs	0.0014	0.04	0.0042	0.12	84
HCB and β-HCH	0.022	0.629	0.066	1.887	89
α-HCH	0.017	0.486	0.051	1.458	91
γ-HCH (Lindane)	0.015	0.429	0.045	1.287	92
DDT	0.012	0.343	0.036	1.029	91
DDE	0.027	0.771	0.081	2.313	94
DDD	0.02	0.571	0.06	1.713	93
Endosulfan (α + β)	0.03	0.857	0.09	2.571	88
Methoxychlor	0.025	0.714	0.075	2.142	91
Dieldrin (Aldrin + Dieldrin)	0.018	0.514	0.054	1.542	92

**Table 2:** Percentage of Samples with ΣPCBs exceeding MRL of 40 ng/g of fat.

	Akkar	Baalbek-Hermel	Batroun	Bekaa	Chouf	Jbeil	Keserwan	Metn	Nabatiyeh	North Area	South Lebanon	ALL Lebanon
ΣPCBs	33.3	40.0	26.7	26.7	53.3	20.0	66.7	13.3	40.0	53.3	73.3	40.6

**Table 3:** Percentage of samples with non-detected PCB Residues.

	Akkar	Baalbek-Hermel	Batroun	Bekaa	Chouf	Jbeil	Keserwan	Metn	Nabatiyeh	North Area	South Lebanon	ALL Lebanon
ΣPCBs	66.7	60.0	73.3	73.3	46.7	80.0	33.3	86.7	60.0	46.7	26.7	59.4

**Table 4:** Regional mean levels of Individual and Total PCB Congeners (ng/g on fat basis) in Dairy Samples Across different Lebanese regions.

	PCB_28_	PCB_52_	PCB_101_	PCB_138_	PCB_153_	PCB_180_	ΣPCBs
Akkar	2.387	2.576	5.949	6.860	9.813	11.387	38.972
Baalbek-Hermel	2.725	3.396	5.260	7.407	9.507	12.280	40.575
Batroun	1.812	2.396	4.363	6.893	8.613	10.113	34.191
Bekaa	2.149	2.850	4.858	6.944	9.472	11.217	37.490
Chouf	2.385	3.349	4.803	8.507	10.800	12.333	42.177
Jbeil	1.879	2.523	4.396	6.853	9.093	10.853	35.597
Keserwan	3.085	4.703	5.289	7.929	12.987	14.160	48.153
Metn	1.679	2.500	3.739	6.100	8.607	9.900	32.524
Nabatiyeh	2.363	3.263	4.980	6.800	8.743	11.180	37.328
North Area	2.432	3.423	5.083	8.867	11.147	12.613	43.564
South Lebanon	2.725	4.489	5.353	9.360	11.483	12.503	45.913
ALL Lebanon	2.329	3.224	4.916	7.502	10.024	11.685	39.680

**Table 5:** Distribution of the thermal power plants across Lebanese regions.

Power Plant Name	Area	Operator	Output
Zouk	Keserwan	EDL	1003 MW
Jieh	Mount Lebanon	EDL	622 MW
Zahrani	South Area	EDL	469 MW
Deir Ammar	North Area	EDL	464 MW
Hreisheh	North Area	EDL	75 MW
Baalbek	Baalbek-Hermel	EDL	70 MW
Tyr (Sour)	South Area	EDL	70 MW

**Table 6:** Regional Distribution of ΣPCB_28,52,101_ and ΣPCB_138,153,180_ and total PCBs in yogurt samples, with 95% Confidence Intervals.

	ΣPCB_28,52,101_	ΣPCB_138,153,180_	Σ of All PCBs	
	Mean ± SEM	Mean ± SEM	95% CI	*p* Value
Akkar	10.91 ± 0.68	28.06 ± 1.24	35.72–42.22	0.508
Baalbek-Hermel	11.38 ± 0.75	29.19 ± 1.68	38.82–45.33	0.799
Batroun	8.57 ± 0.82	25.62 ± 1.68	29.11–39.27	0.028
Bekaa	9.86 ± 0.59	27.63 ± 1.87	33.99–40.99	0.149
Chouf	10.54 ± 0.55	31.64 ± 1.71	37.68–46.68	0.317
Jbeil	8.80 ± 0.71	26.80 ± 1.29	31.50–39.70	0.037
Keserwan	13.08 ± 1.01	35.08 ± 2.34	41.49–54.81	0.02
Metn	7.92 ± 0.68	24.61 ± 1.13	29.06–35.99	<0.001
Nabatiyeh	10.61 ± 0.81	26.72 ± 1.64	32.38–42.28	0.266
North Area	10.94 ± 0.61	32.63 ± 1.69	39.10–48.03	0.109
South Lebanon	12.57 ± 0.76	33.35 ± 2.23	40.12–51.70	0.046
All Lebanon	10.44 ± 0.25	29.25 ± 0.55	38.22–41.16	0.676

**Table 7:** Comparison of the current research with other studies on NDL-PCBs.

Country	Year	Samples	Results	Reference
USA	2010	Frozen yogurt, whole milk	Non-detected (ND) in all samples	[63]
South Korea	2012	Milk, yogurt	0.09 and 0.133 ng/g fat, respectively	[64]
Belgium	2013	Milk samples	4.72 ng/g fat	[65]
Spain	2015	Conventional yogurt and organic yogurt samples	12.55 ng/g fat and 13.21 ng/g fat, respectively	[12]
Romania	2018	Milk samples	9.12 ng/g fat	[66]
Canada and Denmark	2019	Milk and yogurt	1.858 and 1.754 ng/g fat, respectively	[10]
Turkey	2019	Milk samples	127.27 ng/g fat	[67]
Poland	2019	Milk samples	1.25 ng/g fat	[68]
Iran	2023	Yogurt samples	14.65 ng/g fat	[69]
Lebanon	2025	Yogurt samples	39.68 ng/g fat	This study

**Table 8:** Percentage of Samples with OCPs exceeding the corresponding MRL.

	HCB	αHCH	βHCH	γHCH	Endosulfan	Methoxychlor	Dieldrin	ΣDDTs
MRL	10	10	10	10	50	10	6	40
Akkar	86.7	100	86.7	86.7	46.7	80	86.7	100
Baalbek-Hermel	86.7	93.3	93.3	100	86.7	93.3	86.7	100
Batroun	73.3	93.3	80.0	86.7	66.7	86.7	86.7	100
Bekaa	93.3	100	93.3	93.3	93.3	93.3	93.3	100
Chouf	80.0	93.3	93.3	93.3	73.3	80.0	73.3	100
Jbeil	80.0	93.3	86.7	86.7	73.3	80.0	86.7	100
Keserwan	86.7	93.3	80.0	86.7	86.7	86.7	86.7	100
Metn	80.0	93.3	80.0	80.0	46.7	80.0	80.0	100
Nabatiyeh	93.3	93.3	93.3	93.3	86.7	80.0	86.7	100
North Area	86.7	93.3	93.3	93.3	93.3	86.7	86.7	100
South Lebanon	86.7	100	86.7	80.0	100	93.3	100	100
ALL Lebanon	84.9	95.1	87.9	89.1	77.6	85.5	86.7	100

**Table 9:** Percentage of Samples with non-detected OCP residues.

	HCB	αHCH	βHCH	γHCH	Endosulfan	Methoxychlor	Dieldrin	ΣDDTs
MRL	10	10	10	10	50	10	6	40
Akkar	13.3	0	13.3	13.3	53.3	20	13.3	0
Baalbek-Hermel	13.3	6.7	6.7	0	13.3	6.7	13.3	0
Batroun	26.7	6.7	20.0	13.3	33.3	13.3	13.3	0
Bekaa	6.7	0	6.7	6.7	6.7	6.7	6.7	0
Chouf	20.0	6.7	6.7	6.7	26.7	20.0	26.7	0
Jbeil	20.0	6.7	13.3	13.3	26.7	20.0	13.3	0
Keserwan	13.3	6.7	20.0	13.3	13.3	13.3	13.3	0
Metn	20.0	6.7	20.0	20.0	53.3	20.0	20.0	0
Nabatiyeh	6.7	6.7	6.7	6.7	13.3	20.0	13.3	0
North Area	13.3	6.7	6.7	6.7	96.7	13.3	13.3	0
South Lebanon	13.3	0	6.7	20.0	0	6.7	0	0
ALL Lebanon	15.1	4.9	12.1	10.9	22.4	14.5	13.3	0

**Table 10:** OCP Concentration Means (μg/kg on fat basis) across different Lebanese regions.

	HCB	αHCH	βHCH	γHCH	Endosulfan	Methoxychlor	Dieldrin
MRL	10	10	10	10	50	10	6
Akkar	42.31	69.40	57.58	54.30	42.52	20.47	36.17
Baalbek-Hermel	61.64	68.28	75.22	75.60	79.92	40.82	44.83
Batroun	27.75	52.55	53.26	46.22	47.69	26.05	34.77
Bekaa	62.89	69.87	75.75	67.81	91.10	41.49	59.95
Chouf	43.33	55.88	65.49	53.08	59.75	25.54	37.74
Jbeil	34.40	51.28	60.18	49.22	52.06	24.27	36.10
Keserwan	42.58	53.55	60.46	45.22	84.92	25.65	49.30
Metn	34.20	54.22	54.40	45.04	42.42	17.70	34.12
Nabatiyeh	52.75	59.28	74.82	53.88	84.92	29.47	51.97
North Area	53.18	63.28	74.75	60.75	91.43	31.65	52.43
South Lebanon	50.98	61.33	66.58	47.71	96.73	32.36	56.67
ALL Lebanon	45.99	59.90	65.32	54.44	70.32	28.68	46.29

**Table 11:** Mean, SEM, and 95% Confidence Intervals for the various ΣDDTs Concentrations Means (μg/kg on fat basis) across different Lebanese regions.

	DDT	DDE	DDD	ΣDDTs (MRL = 40 μg/kg Fat)	
	Mean ± SEM	Mean ± SEM	Mean ± SEM	95% CI	*p* Value
Akkar	126.88 ± 10.89	159.76 ± 11.87	150.87 ± 6.22	395.42–479.58	<0.001
Baalbek-Hermel	113.34 ± 10.76	185.27 ± 5.50	115.67 ± 10.10	375.59–452.97	<0.001
Batroun	83.69 ± 9.14	146.38 ± 15.89	98.53 ± 3.89	287.37–369.85	<0.001
Bekaa	124.13 ± 5.86	195.33 ± 3.91	155.93 ± 6.23	450.05–500.75	<0.001
Chouf	94.89 ± 10.84	161.78 ± 17.80	117.67 ± 4.65	325.89–422.79	<0.001
Jbeil	84.76 ± 9.87	146.18 ± 15.71	103.53 ± 4.30	291.27–377.68	<0.001
Keserwan	105.69 ± 12.31	149.58 ± 16.07	114.13 ± 4.63	317.35–421.47	<0.001
Metn	74.23 ± 11.12	136.01 ± 18.44	108.00 ± 4.33	278.15–358.34	<0.001
Nabatiyeh	113.89 ± 13.40	170.49 ± 12.67	127.00 ± 6.10	363.39–459.38	<0.001
North Area	97.54 ± 8.00	117.09 ± 13.50	138.20 ± 5.68	376.59–449.09	<0.001
South Lebanon	92.05 ± 16.31	189.47 ± 4.88	134.47 ± 5.96	375.70–456.26	<0.001
All Lebanon	101.01 ± 3.48	165.21 ± 4.18	124.00 ± 2.22	376.79–403.65	<0.001

**Table 12:** Comparative Levels of OCPs (μg/kg on fat basis) in Milk and Yogurt from various countries.

Country	Year	Samples	Results	Reference
Jordan	2009	Cow milk	ΣHCH ^a^: 133; ΣDDTs ^b^: 27; Endosulfan: 30; Dieldrin: ND	[41]
Jordan	2009	Yogurt	ΣHCH: 94; ΣDDTs: 32; Endosulfan: ND; Dieldrin: ND	[41]
Ethiopia	2013	Cow milk (from three different locations)	ΣDDTs: 269; 477; 421	[81]
Ethiopia	2014	Cow milk (from four different locations)	ΣDDTs: 555.02; 263.18; 149.42; 72.52 Endosulfan: 9.56; ND; 15.52; ND	[82]
Spain	2015	Conventional yogurt	ΣHCH: 0.59 ΣDDTs: 7.63 Endosulfan: ND Methychlor: ND; Dieldrin: 7.98	[12]
Spain	2015	Organic yogurt	ΣHCH: 0.74 ΣDDTs: 5.65 Endosulfan: ND Methychlor: ND Dieldrin: 0	[12]
Egypt	2023	Cow milk (from three different locations	ΣDDTs: 232.2; 156.4; 100.4; Dieldrin: 91.3; 95.3; 57.6; γ-HCH: 33.7; 36.9; 52.2	[42]
Lebanon	2025	Yogurt samples	HCB: 45.99; ΣHCH: 179.66 ΣDDTs: 390.22; Endosulfan:70.32; Methoxychlor: 28.68; Dieldrin: 46.29	This study

^a^: sum of α-HCH, β-HCH, and γ-HCH; ^b^: sum of DDE, DDD, and DDT.

**Table 13:** Comparative Levels of OCPs (μg/kg of fresh weight) in Milk and Yogurt from Different Countries.

Country	Year	Samples	Results	Reference
China	2006	Yogurt	ΣHCH: 0.6; ΣDDTs: 0.5	[83]
India	2008	Cow milk	ΣHCH: 162; ΣDDTs: 172.4; Endosulfan: 49.2	[84]
Poland	2013	Cow milk	ΣHCH: 0.72–4.031, ΣDDTs: 1.72–3.75	[85]
China	2017	Cow milk	ΣHCH: 0.63; ΣDDTs: 2.29	[78]
Egypt	2018	Cow milk	ΣHCH: 48.65 ΣDDTs: 54.77 Dieldrin: 13.96	[86]
India	2020	Cow milk samples (from 5; different locations)	HCHs (β-HCH, γ-HCH): 1.11; ND; 0.41; 1.24; 0.17 ΣDDTs: 0.53; 1.60; 0.24; 1.70; 0.60 Endosulfan: 1.24; 0.2; ND; 1.21; ND Methoxychlor: ND; ND; ND; 0.24; ND	[87]
Romania	2020	Cow milk (from 3 different locations	ΣDDTs: 0.181; 0.218; 0.1137	[88]
China	2021	Cow milk	ΣHCH: 0.07; ΣDDTs: 0.1	[89]
Croatia	2024	Cow milk	ΣHCH: 1.04; ΣDDTs: 1.7 Endosulfan: 1.68 Methychlor: 1.78; Dieldrin: 1.1	[43]
Lebanon	2025	Yogurt samples	HCB: 1.61; ΣHCH: 6.29; ΣDDTs: 13.66; Endosulfan: 2.46; Methoxychlor: 1.00; Dieldrin: 1.62	This study

**Table 14:** The estimation of daily intake (EDI) and risk characterization of six PCBs and OCPs in Lebanese artisanal yogurt.

Compound	Mean Value (ng/g Fresh Weight)	HBGV (ng/kg bw/day)	EDI (ng/kg bw/day)	HQ
ΣPCB	1.39	20 ^c^	1.36	0.068
HCB	1.61	170 ^d^	1.58	0.0093
ΣHCH	6.29	2000 ^c^	6.16	0.0031
ΣDDTs	13.66	10,000 ^e^	13.44	0.0013
Endosulfan	2.46	6000 ^e^	2.41	0.0004
Methoxtchlor	1.00	5000 ^f^	0.98	0.0002
Dieldrin	1.62	100 ^e^	1.59	0.0159

^c^ Reference dose [43]; ^d^ Tolerable daily intake—TDI [90]; ^e^ Acceptable daily intake—ADI, and Provisional Tolerable Daily Intake—PTDI [51]; and ^f^ Reference dose [91].

**Table 15:** Comparative Sustainability of Remediation Strategies for Reducing PCBs and OCPs in Dairy Food Chains.

Remediation Method	Energy Demand	Environmental Impact	Dairy Sector Relevance	Circular Economy Potential	Related SDGs	Reference
Bioremediation	Low	Low (natural degradation)	Soil cleanup for feed production	Moderate	SDG 3, SDG 12, SDG 15	[106]
Biochar Adsorption	Low	Very Low (waste-derived)	Water purification for livestock	High	SDG 6, SDG 12, SDG 13	[107]
Phytoremediation	Very Low	Low (plant-based uptake)	Irrigation/fodder field protection	Moderate	SDG 13, SDG 15	[108]
Synergistic Remediation	Low–Moderate	Very Low (integrated methods)	Holistic protection of dairy farming systems	High	SDG 2, SDG 3, SDG 12, SDG 15	[109]

## Data Availability

The original contributions presented in this study are included in the article. Further inquiries can be directed to the corresponding author.

## Abbreviations

The following abbreviations are used in this manuscript:

PCBs	Polychlorinated biphenyls
OCPs	Organochlorine pesticides
MRLs	Maximum Residue Limits
CVD	Cardiovascular Disease
POPs	Persistent Organic Pollutants
SC	Stockholm Convention
HCB	Hexachlorobenzene
α-HCH	Alpha-hexachlorocyclohexane
β-HCH	Beta-hexachlorocyclohexane
γ-HCH	Gamma-hexachlorocyclohexane
DDT	Dichlorodiphenyltrichloroethane
DDE	Dichlorodiphenyldichloroethylene
DDD	Dichlorodiphenyldichloroethane
SPE	Solid-phase extraction
Al_2_O_3_	Aluminum sulfite
H_2_SO_4_	Sulfuric acid
MgSO_4_	Magnesium sulfate
NaCl	Sodium chloride
d-SPE	Dispersive solid-phase extraction
ECD	Electron capture detector
MS	Mass spectrometry
LOD	Limit of Detection
EDI	Estimation of Daily Intake
C	Concentration
MS	Meal Size
bw	body weight
HQ	Hazard quotient
HBGV	Health-Based Guidance Value
ADI	Acceptable Daily Intake
TDI	Tolerable Daily Intake
FAO	Food and Agriculture Organization
WHO	World Health Organization
US EPA United States	Environmental Protection Agency
MW	Megawatt
ASTDR	Agency for Toxic Substances and Disease Registry
NDL-PCB	Non-Dioxin-Like PCB
EFSA	European Food Safety Authority
PTDI	Provisional tolerable daily intake
SDGs	Sustainable Development Goals
